# Improved Estimation of Parkinsonian Vowel Quality through Acoustic Feature Assimilation

**DOI:** 10.1155/2021/6076828

**Published:** 2021-07-14

**Authors:** Amr Gaballah, Vijay Parsa, Daryn Cushnie-Sparrow, Scott Adams

**Affiliations:** ^1^Department of Electrical and Computer Engineering, University of Western Ontario, London, ON, Canada; ^2^School of Communication Sciences and Disorders, University of Western Ontario, London, ON, Canada

## Abstract

This paper investigated the performance of a number of acoustic measures, both individually and in combination, in predicting the perceived quality of sustained vowels produced by people impaired with Parkinson's disease (PD). Sustained vowel recordings were collected from 51 PD patients before and after the administration of the Levodopa medication. Subjective ratings of the overall vowel quality were garnered using a visual analog scale. These ratings served to benchmark the effectiveness of the acoustic measures. Acoustic predictors of the perceived vowel quality included the harmonics-to-noise ratio (HNR), smoothed cepstral peak prominence (CPP), recurrence period density entropy (RPDE), Gammatone frequency cepstral coefficients (GFCCs), linear prediction (LP) coefficients and their variants, and modulation spectrogram features. Linear regression (LR) and support vector regression (SVR) models were employed to assimilate multiple features. Different feature dimensionality reduction methods were investigated to avoid model overfitting and enhance the prediction capabilities for the test dataset. Results showed that the RPDE measure performed the best among all individual features, while a regression model incorporating a subset of features produced the best overall correlation of 0.80 between the predicted and actual vowel quality ratings. This model may therefore serve as a surrogate for auditory-perceptual assessment of Parkinsonian vowel quality. Furthermore, the model may offer the clinician a tool to predict who may benefit from Levodopa medication in terms of enhanced voice quality.

## 1. Introduction

Parkinson's disease (PD) is the second most common neurodegenerative disease, after Alzheimer's disease [1]. Pathological symptoms of PD are severe loss of dopaminergic neurons in the nigrostriatal region and the appearance of cytoplasmic inclusions known as Lewy bodies (LBs) [2, 3]. A reduction in dopamine production leads to the appearance of rest tremors, akinesia, cogwheel rigidity, and postural instability. In addition, statistics show that nearly 90% of people impaired with PD develop voice and speech disorders during the course of their disease [4, 5]. The classic characteristics of Parkinsonian speech and voice include reduced vocal loudness (hypophonia), with a tendency of the voice to fade out; reduced prosodic pitch inflection (hypoprosodia); breathy or hoarse voice; imprecise articulation of consonants and vowels; and mumbled speech [4, 5]. While speech articulation and fluency problems appear at later stages of PD, voice abnormalities may appear at earlier stages of the course of the disease [5]. As such, assessment of voice characteristics of PD patients forms a critical part of their treatment and rehabilitation processes.

For medical treatment, Levodopa is the most commonly used medication that has been shown to improve the motor symptoms of the disease [6]. Levodopa crosses the blood-brain barrier and increases the production of dopamine. This process reduces the effect of the dopamine production drop caused by PD and enhances the efficiency of the motor features of the PD subject [6]. Despite its therapeutic effects in the treatment of motor deficits of PD, Levodopa does not have the same healing effect on PD voice. In general, the magnitude, consistency, and long-term effects of Levodopa are far from satisfactory for voice rehabilitation in PD patients [5, 7]. For example, Cushnie-Sparrow et al. [8] recently investigated the effect of the Levodopa on the perceived quality of vowels produced by PD patients. Vowel samples were collected from 51 PD subjects before and after Levodopa administration, and these samples were rated by three listeners. Results showed no statistically significant difference in the perceived quality of the vowels produced by PD patients before and after taking the Levodopa. A more in-depth analysis revealed an interesting fact: there was a statistically significant *improvement* in the vowel quality with the administration of Levodopa for those PD patients whose off-medication vowel quality was rated as poor. This finding motivates the need for the assessment of vowel samples from PD patients so that the potential benefit from Levodopa medication on their voice quality can be estimated.

While subjective assessment is considered the gold standard of voice quality evaluation, it is not efficient in terms of time and cost, especially when multiple voice samples need to be rated by a group of listeners [9]. This weighs in favour of objective, instrumental assessment of voice quality [9]. Traditional acoustic characterization of vowel samples includes jitter, shimmer, harmonics-to-noise ratio (HNR), and cepstral peak prominence (CPP). Jitter is defined as the cycle-to-cycle variation of the fundamental frequency, while the relative jitter is the ratio between the absolute jitter and the average fundamental frequency [10]. Shimmer is defined as the variability of the peak-to-peak amplitude in decibels, while relative shimmer is the ratio between the absolute shimmer and the average amplitude [10]. HNR quantifies the relationship between the periodic components (harmonics) and the aperiodic components (noise) of the signal [8]. Finally, CPP is defined as the difference between the peak of the cepstrum and its linear regression function [11].

Few studies investigated the performance of traditional acoustic measures in predicting the perceived quality of PD vowels. Jannetts et al. [12] collected recordings of the sustained phonation of /a/, a sentence, and normal conversation from 43 speakers with PD and 10 participants with ataxia. The recordings were rated subjectively using the GRBAS scale (G: the grade or overall dysphonia severity, R: roughness, B: breathiness, A: asthenia or weakness, and S: strain). Among the aforementioned traditional acoustic measures, CPP was found to produce the highest Spearman's rank-order correlation with “Grade” voice quality attribute.

Cushnie-Sparrow et al. [8] investigated the effect of the Levodopa medication on the perceived quality of vowels produced by PD patients. Sustained vowel recordings were collected from 51 subjects impaired with PD, in addition to 11 healthy control individuals. Measured acoustic metrics included jitter, shimmer, HNR, CPP, and the acoustic voice quality index (AVQI). Measurement of the perceived quality was obtained from a panel of 3 listeners. The authors found that the HNR resulted in the highest Pearson's correlation coefficient of 0.55 with the averaged subjective quality scores.

In addition to these traditional measures, a multitude of other features has been extracted from vowel samples based on nonlinear dynamic, filterbank, and spectrotemporal modulation analyses (e.g., [13, 14]). Examples of such features include the recurrence period density entropy (RPDE), Mel and Gammatone frequency cepstral coefficients (MFCCs and GFCCs), linear prediction (LP) based features, and modulation spectrogram features. In addition, these features often need to be combined through a linear or nonlinear regression model, so that a single index of disorder severity may be obtained. A vast majority of past studies investigating these features and feature mapping models focused on their effectiveness in discriminating between normal and PD vowel samples (e.g., [14]) or in predicting the Unified Parkinson's Disease Rating Scale (UPDRS) [13]. To the best of our knowledge, no study has investigated the application of these features and their regression models in predicting the perceived quality of PD vowels.

This work, therefore, aims to build a valid regression model that assimilates relevant acoustic features for improved estimation of the perceived quality of PD vowels. Using the previously collected subjective database by Cushnie-Sparrow et al. [8], the performance of several acoustic features was assessed, both individually and in combination. Multivariate linear regression and support vector regression techniques were utilized to assimilate the feature sets, both with and without feature reduction techniques. A final composite objective index was developed that produced a statistically significant improvement in predicting the perceived PD vowel quality ratings.

## 2. Methods

### 2.1. Voice Recordings and Subjective Evaluation

As mentioned earlier, subjective data collected by Cushnie-Sparrow et al. [8] were used to develop and benchmark the performance of the objective metrics. A brief description of the subjective data collection procedure is given here for the sake of completion. Samples of the sustained vowel ‘ah' were collected from 51 PD subjects. Salient demographic data includes (a) 39 male and 12 female subjects, (b) age range of 47 to 82 years (*M* = 65.78, SD = 4.19), (c) diagnosis duration range of 2 to 16 years (*M* = 9.22, SD = 4.19), and (d) Levodopa use duration range of 2 to 16 years (*M* = 7.51, SD = 3.91) [8].

PD patients were evaluated off and on the Levodopa medication. In addition, sustained vowel recordings were also collected from 11 subjects who were nonimpaired with PD; these recordings served as a control of the measurement process. All recordings were collected using a high-quality headset microphone (DPA 4060) at a 44100 Hz sampling rate and 16 bits/sample quantization. The headset microphone was placed 6 cm horizontal from the middle of the upper lip philtrum and to the side of the mouth (approximately 45 degrees). Speech intensity was calibrated using a 70 dB SPL reference and a sound level meter positioned 15 cm from the mouth of the PD patient. A total of 113 vowel recordings were collected through this procedure. Two-second samples from the middle of each vowel recording were extracted for analysis, and perceptual judgments of each segment were provided by 3 listeners (graduate students in the Speech Language Pathology program at Western University). The listeners were instructed to judge the *overall* voice quality of each vowel sample using a visual-analogue scale (VAS). The scale was 10 cm in length, and the endpoint descriptors were “poor voice quality” on the left and “better voice quality” on the right. VAS score was recorded as the distance from the left endpoint to the listeners' mark. The average of the three listener ratings served as the overall quality rating of the vowel recordings. To test intrarater reliability, 20% of the vowel samples were randomly selected and inserted into the presentation order in a random fashion, for a rerating. More details can be found in [8].

### 2.2. Features and Their Computation

Subjective ratings obtained through the procedure outlined in the previous section were used to benchmark the performance of the objective measures. Prior to feature extraction, the sustained vowel recordings were decimated to a 16 kHz sample rate. In addition, the complete 113-sample database was divided into 2 datasets. The first dataset contained 80% of the whole dataset or 91 samples to constitute the training dataset, while the remaining 20% of the data or 22 voice samples made up the test dataset.

#### 2.2.1. Filterbank-Based Features

GFCC coefficients are mainly used in computational auditory sense analysis (CASA) studies to transform signals into time-frequency (T-F) domain to perform robust speech recognition [3, 15]. The recorded signal was segmented into frames of 256 samples, with a frame overlap of 100 samples. Afterwards, the power spectrum of each frame was obtained after multiplying with a Hamming window. The equivalent rectangular bandwidth (ERB) filterbank was applied to the frame power spectra. In this research, 128 filters constituted the ERB filterbank, and the log filterbank energies were decorrelated using the discrete cosine transform (DCT) [15]. The frame averaged GFCCs and their first-order time differences (“delta” values) resulted in the final GFCC feature set that contained 60 features.

#### 2.2.2. Modulation-Based Features

Two features extracted from the envelope, namely, the speech-to-reverberation modulation energy ratio (SRMR) and modulation area (ModA), served as the modulation-based features [9]. The envelope of the waveform was extracted and filtered to a number of filters [16, 17]. The ratios of the energy in the low band filters, which are assumed to contain the speech modulation energies, and the high band filters which are assumed to contain the noise modulation energies represent the quality of the sustained vowel signal.

In SRMR [16], the speech signal was processed through a 23-channel Gammatone filterbank with center frequencies ranging from 125 Hz to half the sampling rate. Hilbert transform was then applied to the filterbank outputs, to extract the temporal envelope in each channel. These envelopes had frequencies that ranged between 0 and 128 Hz. At this point, each envelope was filtered into eight overlapping modulation bands, with center frequencies ranging from 4 to 128 Hz. Finally, SRMR was computed as a ratio between the energy stored in the first four filters, which contain most of the speech energy, and the last four filters, which contain the background noise [16].

In ModA, the speech signal was decomposed using only 4 bandpass filters, and filtered signals had Hilbert transform applied to derive the band-specific temporal envelopes. Each envelope was subsequently downsampled to 20 Hz and then processed through 1/3 octave filterbank with center frequencies ranging between 0.5 and 8 Hz. The filterbank output energies were then used to derive the area under each acoustic band, and then those areas are averaged to produce the ModA metric [17].

#### 2.2.3. Linear Prediction-Based Features

The LP-based feature extraction methodology is presented in Low Complexity Quality Assessment (LCQA) proposed by Grancharov et al. [18]. The central idea of LCQA is to extract statistical features of the speech signal [18]. Each speech recording was segmented into 20 ms nonoverlapping frames, an 18^th^ order LP model was computed for each frame, and a vector of features is extracted from each frame. This features' vector incorporates 10 features which are the spectral flatness, the excitation variance, the signal variance, the spectral centroid, and the spectral dynamics for each frame in addition to the first derivative of each of the aforementioned features [19]. At this point, the statistical properties of each one of the 10 features are calculated across all the frames; these statistical features include mean, variance, skew, and kurtosis [18]. This yields to the formation of a vector of size 40 × 1 for each speech signal [15, 19].

#### 2.2.4. Recurrence Period Density Entropy (RPDE)

In RPDE measurements, the signal was first applied to a time delay embedding to recreate the phase space of a nonlinear dynamic system [20]. RPDE quantifies the percentage of the dynamics in the reconstructed phase space that are periodic or repeated exactly [20, 21]. Recurrence time (*T*) is the time that the recurrent signal takes to turn back to the same point [20]. It was previously shown that the deviation from the entropy calculated by the entropy *H* of the distribution of these recurrence periods is a good indication of general voice disorders [20]. RPDE has been used in [20] to classify disordered voice and normal voice, and its accuracy reached 91%. These results led to incorporating RPDE in this study to assess the quality of Parkinsonian sustained vowels. The RPDE was computed using the voice analysis toolbox based on the research by Tsanas et al. [13].

#### 2.2.5. Traditional Acoustic Measures

The traditional acoustic measures of percent jitter, absolute shimmer, HNR, and CPP was computed from the sustained vowel records using the Praat software package (version 6.0) [22]. The records were analyzed using a custom Praat script, and the traditional acoustic measures were extracted from the report of voice characteristics returned by the script.

### 2.3. Feature Mapping

While RPDE, HNR, and CPP are single numbers that represent the predicted speech quality, the GFCC and LCQA are multidimensional feature vectors. Mapping algorithms aim to generate a function that assimilates the multidimensional feature vectors to match the subjective scores. To express this mathematically, we have [23]

(1) $$\begin{array}{c} y=f\left(\theta ,X\right)+b, \end{array}$$

where *θ* represents the parameters and functions associated with the feature mapper, **X** is the feature matrix that has size *m* × *n*, *m* is the number of training samples, *n* is the size of the feature vector, **y** are the subjective scores corresponding to the training samples, and **b** is the prediction error. Commonly used feature mappers include multivariate linear regression (LR) and support vector regression (SVR) [24].

### 2.4. Principal Component Analysis (PCA)

PCA is used to reduce the dimensionality of the input features of the machine learning algorithm and enhance the interpretation of the features [25]. This dimensionality reduction or feature reduction has to be done in a way that maintains the information contained in the input features [25]. PCA utilizes the eigenvalues and the eigenvectors to come up with new features that have smaller dimensionality but maximizes the variance of the dataset [25]. More details about PCA can be found in [25].

### 2.5. Feature Selection and Reduction

A higher dimensionality of the feature vector may cause overfitting. In such situations, extracted numbers of features for each metric must be reduced before applying the machine learning algorithm to avoid overfitting. To accomplish this goal, the correlation between each single feature and the subjective scores was obtained, and then the features were rearranged according to their correlation values from the highest to the lowest. Subsequently, a Monte Carlo algorithm was applied to extract the maximum number of features that minimized the cost function for both the training and the test datasets. This algorithm took the rearranged features' matrix and the subjective scores vector as two inputs [26]. At this point, the data was split into a training dataset and test dataset where the training dataset contained 80% of the full data, while the test dataset contained the remaining 20% of the dataset. The algorithm applied linear regression to a subset of the datasets to find which subset achieved the minimum mean square error (MSE) with the subjective scores.

## 3. Results

### 3.1. Subjective Results

Intrarater reliability of the perceptual judgment of voice quality was assessed using the intraclass correlation coefficient (ICC) [8]. Each rater was assessed using average agreement in a two-way mixed model. The average ICC across all raters [8], which is considered to be moderate intrarater reliability. Interrater reliability across the 3 subjective estimators was assessed using average consistency in a two-way random model, average [8], which can be interpreted as good interrater reliability.

**Figure 1:**
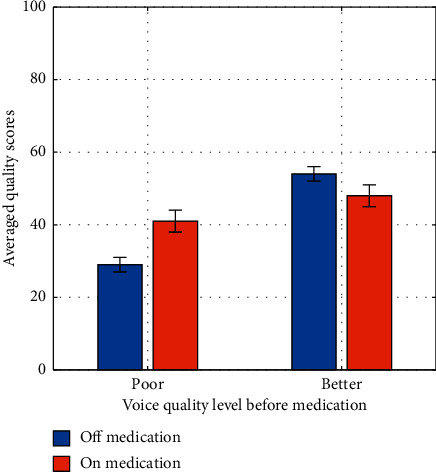
Averaged perceptual overall vowel quality scores off and on medication for the two PD groups: those whose premedication quality score was below a threshold (labeled as “poor” quality level) and those with a score above the threshold (labeled as “better” quality level). The threshold was set as the lower 95% confidence interval for the mean vowel quality rating of the control subjects.

Paired sample *t*-tests showed that there were no statistically significant differences between PD vowel quality ratings on and off Levodopa. In other words, when the PD patient cohort was considered as a whole, the PD medication did not have any influence on their vowel quality. An interesting finding does emerge, however, when PD group is divided into two groups: those with poor perceived voice quality and those with good perceived voice quality in the off-medication condition. There was a significant improvement in perceived vowel quality for the poor-quality group with the administration of medication. The differences among the two groups in terms of the off-medication voice quality and the improvement after medication are shown in Figure 1. It can be seen that patients who have low sustained vowel quality ratings before taking Levodopa have a high improvement in voice quality after taking the medication. On the other hand, people who have high voice quality ratings before taking the medication have a statistically insignificant change in voice quality. These results highlight the need for either subjective or objective assessment of PD voice quality, in order to predict the effectiveness of Levodopa medication on voice quality.

### 3.2. Objective Results

**Figure 2:**
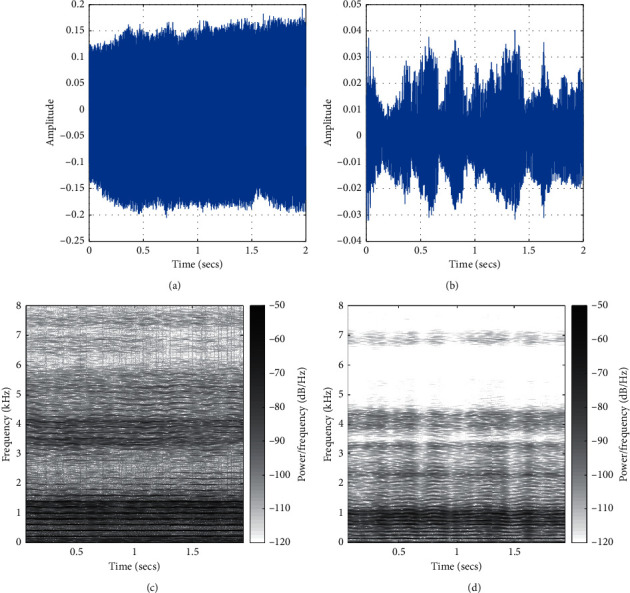
Waveforms and spectrograms of selected sustained vowels recordings from subjects in the database. (a, c) The waveform and the spectrogram for the sustained vowel “∖*a*∖” collected from a normal control subject; (b, d) the waveform and the spectrogram for “∖*a*∖” sustained vowel collected from a PD subject who has been off his medication. (a) Control subject waveform. (b) PD subject waveform. (c) Control subject, high quality. (d) Parkinsonian subject, poor quality.

**Figure 3:**
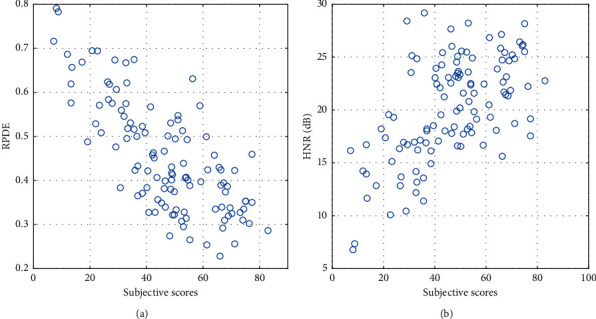
Scatter plots of subjective scores against recurrence period density entropy (RPDE) and harmonics-to-noise ratio (HNR) for the whole dataset. The *x*-axis on these plots is the averaged subjective score across the whole database, while the *y*-axis represents the acoustic measure. The correlation coefficient between the objective and subjective measures is presented in the text. (a) RPDE. (b) HNR.

Figure 2 displays the sample spectrograms associated with sustained vowel samples collected from 2 subjects in the database.  Figure 3(b) displays the spectrogram of the normal control subject with a high subjective quality score. This record had a relative jitter of 0.29%, a relative shimmer of 2.99 dB, a CPP value of 11 dB, and a HNR value of 22.8 dB. Figure 3.2 displays the spectrogram of a subject impaired with PD. This subject had been off Levodopa medication and had a low sustained vowel quality rating. This record of the PD subject has a relative jitter of 1.02%, a relative shimmer of 12.13 dB, a CPP value of 15.5 dB, and a HNR value of 14.23 dB.

Detailed analyses revealed that jitter and shimmer had poor correlation values with the subjective scores of the quality of sustained vowel records, and therefore, they were not considered to be reliable objective metrics of the quality of Parkinsonian sustained vowels.

Table 1 shows (a) the correlation values between the objective scores and the subjective perceived quality ratings using different metrics, and (b) standard deviation of prediction error (SDPE) given by $\text{SDPE}={\hat{\sigma }}_{s}\sqrt{1-\rho^{2}}$, where ${\hat{\sigma }}_{s}$ is the standard deviation of the subjective speech quality scores and *ρ* is the correlation coefficient between the true and predicted quality scores [27]. The statistical significance of the *ρ* parameter was computed as well, and correlation coefficients with significance values *p* < 0.05 and *p* < 0.01 are denoted by ∗ and ∗∗, respectively. It must be noted here that while high correlation coefficients between objective and subjective measures are desirable, a big difference between the correlation coefficients for training and test datasets is an indication of overfitting.

1. *Individual Feature Performance*. The first five objective metrics, SRMR, ModA, CPP, HNR, and RPDE, are solo features. Consequently, LR was applied to the training dataset which contained 80% of the whole database, and then the trained linear model was applied to the test dataset to benchmark the objective metric performance. Both SRMR and ModA had low, statistically insignificant correlation values with the quality of sustained vowels for the training and the test datasets. An explanation of that is the envelope of the high-quality sustained vowel does not have a lot of variations, while the envelope of the low-quality sustained vowel contains high variations (see Figures 3(a) and 3(b), respectively). This contradicts the way SRMR and ModA measure the quality of running speech in which the variations of the envelope and the ratio between energies in the low band and the energies in the high bands of the envelope indicate the quality of the waveform. The correlation between HNR and the subjective scores was 0.55 for the training database and 0.74 for the test database, while the correlation values between the subjective measurements and the CPP scores were 0.29 and 0.53 for the training and the test datasets, respectively—all of which were statistically significant. RPDE was the highest among the single-feature objective metrics to have values of correlation with the subjective scores that reached statistically significant 0.80 and 0.75 values for the training and the test datasets, respectively. It is noted that there is still a difference between the correlation values of the training and the test datasets of RPDE, which means that this metric has deficiency in predicting the quality for new (i.e., unseen) sustained vowel samples.  Figure 3 shows the scatter plot for the RPDE and HNR measures against the subjective scores.
2. *Objective Metrics with Multiple Features*. The full set metrics are the metrics that contained multiple features and the whole set of features are trained without any feature reduction. Using machine learning algorithms on the GFCC features did not yield a reliable metric to estimate the Parkinsonian voice quality while applying SVR on the LCQA features resulted in a LCQA-SVR metric that has a 0.66 correlation value with the subjective scores.
3. *Reduced Multiple Feature Objective Metrics*. Applying PCA and the Monte Carlo feature selection and reduction method to the GFCC and LCQA features led to a reduction of the number of dimensions of the GFCC metric from 60 to 3 features only. It also led to reducing the number of LCQA features from 40 to 16. It is noted that the metrics resulting from the feature reduction method led to higher performance than the PCA method. This enhanced the performance of most of the metrics. Applying LR to the LCQA metric led to obtaining an objective metric that has a statistically significant 0.75 correlation value with the subjective scores.
4. *A Composite Objective Voice Quality Estimator*. A composite metric was derived by augmenting the HNR and CPP features with LCQA features and applying SVR and LR to estimate the vowel quality scores. The combined metric, which included 42 features, resulted in predicted quality scores that had a 0.77 correlation value with the subjective quality scores. This is noteworthy in that it is higher than all the other multiple feature metrics.

**Figure 4:**
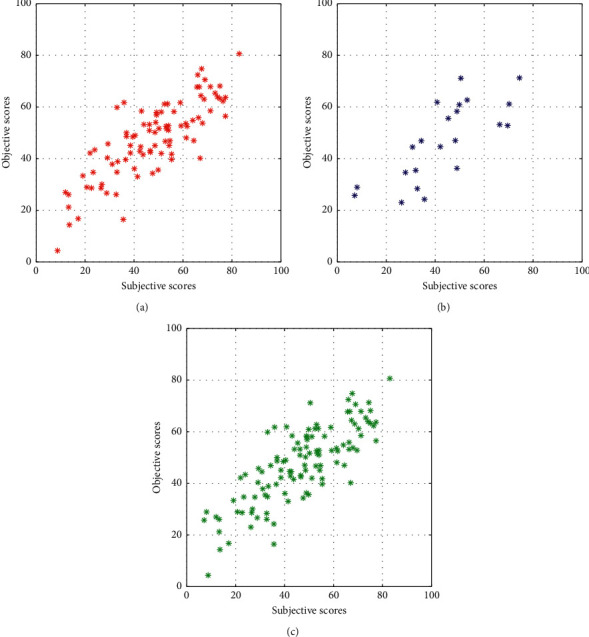
Subjective scores versus objective scores using the reduced combined linear regression (LR) metric. (a) shows the scatter plot for the training data, (b) shows the scatter plot for the test data, and (c) depicts the scatter plot for the whole database.

Afterwards, the PCA method was applied to the features before training the model to estimate the vowel quality. The number of dimensions was reduced to 23 features, which explained 95% of the data variance. Finally applying the feature reduction method had greater improvement of the performance more than using PCA. Applying LR to the reduced combined feature set resulted in a model that estimated the quality of the vowels with a statistically significant 0.80 correlation value with the subjective scores. To test the statistical significance between the correlation values of the obtained scores from the combined metric compared to the second-highest objective metric which is the reduced LCQA metric, Steiger's *Z*-test [28] was applied to measure the statistical difference. It was found that there is a statistical enhancement when using the combined reduced metric instead of the reduced LCQA metric.  Figure 4 shows the scatter plot of the subjective voice quality scores on the *x*-axis against the predicted quality scores by the composite metric on the *y*-axis for the training, test, and full data.

It is noted that the composite metric had the highest correlation for training and test datasets, followed by the RPDE feature. In order to further confirm this finding, both these metrics were trained repeatedly with different training datasets (different samples selected randomly from the whole dataset each time) and then applied to the corresponding test dataset. Then, the average correlation values for the training and the test datasets were calculated, and they were found to be 0.75 for the RPDE method and 0.80 for the combined reduced method. The difference between these two correlation coefficients was statistically significant, indicating the composite measure provided a better overall performance that was robust to random partitioning of the database.

## 4. Discussion and Conclusion

In this paper, the quality of the sustained vowels produced by patients with PD was predicted through objective acoustical analyses. A previously collected vowel database by Cushnie-Sparrow et al. [8] was utilized for this purpose. This database consisted of sustained vowel samples from 51 PD patients before and after taking the Levodopa medication, along with vowel samples from 11 healthy control subjects, which resulted in the formation of a database of 113 vowels. A panel of 3 listeners rated the perceived quality of these vowel recordings [8], which were used in training the objective models and assessing their accuracy. Vowel quality prediction features included GFCC, LCQA, HNR, smoothed CPP, and RPDE. Machine learning algorithms SVR and LR were applied to these multidimensional features to estimate the quality objectively. Some of the features mentioned above were blended to form a composite objective metric that displayed significantly better performance than the other metrics. Moreover, PCA and feature reduction were applied to reduce the number of input features to machine learning algorithms to reduce the overfitting and enhance the performance of the objective metrics.

Initial investigation focused on individual features and parameters extracted from the vowel samples. Although a number of these individual features exhibited statistically significant correlation values with auditory-perceptual ratings, only a subset of the measures exhibited correlation coefficients greater than 0.5. Of the commonly reported vowel acoustic measures, HNR performed the best, with Pearson correlation coefficients of 0.55 and 0.74 for the training and test partitions, respectively. The correlation coefficients exhibited by HNR and CPP [8] but are lower than those reported by Jannetts and Lowit [12]. It is worthwhile to note that Jannetts and Lowit [12] reported Spearman's rank-ordered correlation between the perceptual ratings and acoustic measures, unlike Pearson's correlation coefficient reported here, which can perhaps explain the discrepancy. Furthermore, Jannetts and Lowit [12] employed the GRBAS scale, and the auditory-perceptual ratings were provided by an experienced clinician. It is plausible that these methodological differences also contributed to the differences.

Among the individual measures, the RPDE parameter produced the best performance. The correlation coefficients of 0.8 and 0.75 with training and test partitions were significantly better than those reported by other individual measures. RPDE has been previously employed for discriminating between normal and PD voices [14, 20], and our results demonstrate that it is suitable for predicting the perceived quality of PD vowel samples.

While GFCC was used in other studies to measure the quality of Parkinsonian speech and had a good performance [15, 26], this was not the case for estimating the perceived quality for Parkinsonian sustained vowels. The best performance for GFCC objective metric after feature reduction resulted in a 0.55 correlation coefficient between the subjective and the objective scores. For the nonreduced full set category, applying SVR to the combination of the 40 LCQA feature, HNR, and smoothed CPP was the best objective metric of this category with a correlation value of 0.77 for the test dataset. The difference between the training and the test dataset was the minimum, which meant that the effect of overfitting is minimum. Applying PCA to the features led to the enhancement of most of the objective metrics. It is noted that using LR and SVR on the PCA reduced combined metric yields to statistically similar results. Applying the feature reduction method to the objective features yielded a great enhancement in the performance of the objective metrics. Applying LR and SVR to the reduced combined metric yielded statistically similar results. However, the metric with linear regression had a smaller difference between the training and the test datasets which means it is less prone to overfitting. As a result, the reduced composite metric with linear regression is considered to be the best objective metric for estimating the quality of the Parkinsonian sustained vowels.

In summary, a subset of acoustic measures including HNR, LCQA, and RPDE exhibited a good correlation with auditory-perceptual voice assessments of the overall quality of sustained vowels produced by a group of Parkinson's patients. The application of a regression model (LR and SVR) incorporating a subset of these acoustic features resulted in a statistically improved prediction of the perceived quality of the Parkinsonian vowels. The subjective ratings used for benchmarking the objective models were obtained from sustained vowels produced by PD patients both on and off Levodopa medication. As such, the clinical implications of the current study include the following: (a) the derived model may serve as a surrogate for the subjective assessment of the effect of the Levodopa medication on the voice quality of Parkinsonian subjects. Since the evidence shows that Levodopa improves the voice quality of Parkinsonian patients only when their premedication voice quality is poor, the derived model can potentially play a clinically relevant role in predicting the effectiveness of Levodopa medication. (b) More generally, the derived model can potentially be applied for clinical assessment of the perceived quality of Parkinsonian vowels, particularly for monitoring vowel quality over the course of any therapeutic intervention.

Before closing, a few limitations of our study must be acknowledged. The auditory-perceptual ratings used to train and benchmark models were garnered from clinical graduate students with little experience. Follow-up research focusing on model performance with auditory-perceptual ratings from experienced clinicians is necessary. While the derived model is promising for objective, instrumental assessment of Parkinsonian vowel quality, future research is warranted to test its robustness and generalization capabilities and to further improve its performance. One of the aspects that need to be addressed is the limited size of the collected dataset used in this investigation. The size of the dataset needs to be increased to ensure more reliability and generalization capability of the proposed metrics. Another area for future research is to develop and evaluate more advanced and complicated machine learning algorithms such as deep learning. Applying deep learning emphasizes the need for a larger dataset that needs to be collected to present a more reliable and precise metric to estimate the sustained vowels' quality. The effect of gender on studied acoustic variables is also of future research interest, especially on establishing the effectiveness of the derived model in predicting male versus female PD patient vowel quality. Finally, expanding the findings of this study to estimate the quality of continuous speech (as opposed to sustained vowel) will be of broad research interest.

## Figures and Tables

**Table 1:** Correlation coefficients and standard deviation of prediction error (SDPE) of objective metrics with subjective scores. Indices that are bolded in a column are statistically similar in their performance but are significantly better than others in that column. Significance of correlation values indicated by ^*∗*^*p* < 0.05 and ^*∗∗*^*p* < 0.01.

Metric	Full set				PCA				Reduced			
	Correlation (training)	SDPE (training)	Correlation (test)	SDPE (test)	Correlation (training)	SDPE (training)	Correlation (test)	SDPE (test)	Correlation (training)	SPDE (training)	Correlation (test)	SDPE (test)
SRMR	0.28^*∗∗*^	17.26	0.16	17.74	—	—	—	—	—	—	—	—
ModA	0.40^*∗∗*^	16.48	0.33	16.97	—	—	—	—	—	—	—	—
CPP	0.29^*∗∗*^	17.20	0.53^*∗*^	15.24	—	—	—	—	—	—	—	—
HNR	0.55^*∗∗*^	15.01	**0**.**74**^*∗∗*^	12.09	—	—	—	—	—	—	—	—
RPDE	0.80^*∗∗*^	10.79	**0**.**75**^*∗∗*^	11.98	—	—	—	—	—	—	—	—
GFCC-LR	0.86^*∗∗*^	9.15	−0.25	17.44	0.65^*∗∗*^	13.63	0.42	16.35	0.56^*∗∗*^	14.86	0.55^*∗∗*^	15.05
GFCC-SVR	0.60^*∗∗*^	14.35	0.06	17.98	0.60^*∗∗*^	14.35	0.30	17.19	0.54^*∗∗*^	15.10	0.52^*∗*^	15.39
LCQA-LR	0.82^*∗∗*^	10.27	0.46^*∗*^	16.00	0.76^*∗∗*^	11.66	0.55^*∗∗*^	15.05	0.75^*∗∗*^	11.87	**0**.**75**^*∗∗*^	11.92
LCQA-SVR	0.66^*∗∗*^	13.48	0.66^*∗∗*^	13.54	0.73^*∗∗*^	12.26	0.53^*∗*^	15.28	0.66^*∗∗*^	13.48	0.66^*∗∗*^	15.28
Combined-LR	0.87^*∗∗*^	8.85	0.51^*∗*^	15.50	0.73^*∗∗*^	12.26	0.70^*∗∗*^	12.87	0.81^*∗∗*^	10.52	**0**.**80**^*∗∗*^	10.81
Combined-SVR	0.75^*∗∗*^	11.87	**0**.**77**^*∗∗*^	11.50	0.71^*∗∗*^	12.63	0.66^*∗∗*^	13.54	0.71^*∗∗*^	12.63	**0**.**82**^*∗∗*^	10.31

PCA: principal component analysis; RPDE: recurrence period density entropy; SRMR: speech-to-reverberation modulation ratio; HNR: harmonics-to-noise ratio; CPP: cepstral peak prominence; ModA: modulation area; GFCC: Gammatone frequency cepstral coefficients; LCQA: Low Complexity Quality Assessment; and SVR: support vector regression.

## Data Availability

The data used to support the findings of this study are restricted by the ethics board at Western University in order to protect patient privacy.
